# THE RELATIONSHIPS BETWEEN INTRINSIC CAPACITY AND FRAILTY AND DISABILITY IN OLDER OUTPATIENTS

**DOI:** 10.1093/geroni/igad104.3658

**Published:** 2023-12-21

**Authors:** Mei-Ching Chang, Yu-Tai Lo, Wei Lee, Sheng-Yu Fan

**Affiliations:** National Cheng Kung University, Tainan, New Taipei, Taiwan (Republic of China); National Cheng Kung University, Tainan, New Taipei, Taiwan (Republic of China); National Cheng Kung University, Tainan, New Taipei, Taiwan (Republic of China); National Cheng Kung University, Tainan, New Taipei, Taiwan (Republic of China)

## Abstract

The ICOPE scale is developed by the WHO to assess intrinsic capacity, including cognition, mobility, nutrition, hearing, vision, and depression. However, the relationships between intrinsic capacity and frailty and disability were unknown. The purposes of this study were to explore the cut-off points of the ICOPE scale for frailty and disability in older outpatients, and the significant intrinsic capacities for frailty and disability. This study employed a cross-sectional research design. The participants were patients aged 65 and above attending the geriatric department of a medical center (n=397). The ICOPE scale, Clinical Frailty Scale (CFS), and Activity of Daily Living (ADL) scale were used. ROC curve analysis was used to determine the optimal cut-off points of the ICOPE scale for frailty and disability, and logistic regression for the significant intrinsic capacities related to frailty and disability. For frailty, the AUC value of abnormal intrinsic capacity was 0.79 (p < 0.001), with an optimal cut-off point of 2/3. For disability the AUC value of abnormal intrinsic capacity was 0.82 (p < 0.001), with an optimal cut-off point of 2/3. Logistic regression showed cognition, mobility, and nutrition related to frailty significantly; whereas cognition, mobility, nutrition, and hearing related to disability significantly. Older adults with three or more abnormal intrinsic capacities have a higher risk of frailty and disability. The problems of cognition, mobility, and nutrition were the risk factors of frailty and disability. Healthcare staff can pay attention to older outpatients with the problems of intrinsic capacities.

